# Clinical features and long-term prognosis of patients with congestive heart failure taking tolvaptan: a comparison of patients with preserved and reduced left ventricular ejection fraction

**DOI:** 10.1007/s00380-021-01957-1

**Published:** 2021-10-14

**Authors:** Toshiki Seki, Yoshiaki Kubota, Junya Matsuda, Yukichi Tokita, Yu-ki Iwasaki, Wataru Shimizu

**Affiliations:** Department of Cardiovascular Medicine, Nippon Medical School, 1-1-5 Bunkyo-ku, Sendagi, Tokyo, 113-0022 Japan

**Keywords:** Congestive heart failure, Left ventricular ejection fraction, Long-term prognosis, Tolvaptan

## Abstract

Few studies have investigated the clinical benefit of the long-term use of tolvaptan (TLV) for heart failure (HF). This study evaluated the long-term prognosis of patients administered TLV for > 1 year among patients who had HF with preserved ejection fraction (HFpEF) and those who had HF with reduced ejection fraction (HFrEF). Overall, 591 consecutive patients were admitted to our hospital and administered TLV for HF between 2011 and 2018. We retrospectively enrolled 147 patients who were administered TLV for > 1 year. We divided them into the HFpEF group (*n* = 77, 52.4%) and the HFrEF group (*n* = 70; 47.6%). Their clinical backgrounds and long-term prognosis were examined. Compared with the patients in the HFrEF group, the patients in the HFpEF group were significantly older and included more women. Moreover, the HFpEF group showed significantly lower all-cause mortality (38.6% vs. 24.7%; log-rank, *P* = 0.014) and cardiovascular mortality during the average 2.7-year follow-up. Univariate analysis revealed that all-cause mortality was correlated with male sex, HFpEF, and changes in serum creatinine levels from baseline. Multivariate analysis revealed that HFpEF was an independent influencing factor for all-cause mortality (hazard ratio, 0.44; 95% confidence interval, 0.23–0.86; *P* = 0.017). Long-term administration of TLV may be more beneficial for HFpEF than for HFrEF.

**Supplementary Information:**

The online version contains supplementary material available at 10.1007/s00380-021-01957-1.

## Introduction

The prevalence of heart failure (HF) continues to rise in developed countries [1]. Japan has one of the highest proportions of aged persons in the world and its population of HF patients is progressively increasing, despite the country’s depopulation. HF is expected to affect 1.32 million patients by 2035 [2]. The high readmission rate for HF is a problem that should be resolved. Past real-world data from Japan in 2015 had indicated that the 1-year mortality of HF was 23% and that the 1-year readmission rate was 26.2% [3]. Compared with patients with no or a single previous HF admission, HF patients with multiple previous HF admissions had a significantly higher risk of all-cause death and HF readmission within 3 years [4]. To reduce readmission for HF and mortality, clinicians have to provide appropriate medical therapy to HF patients.

Tolvaptan (TLV) is an oral selective vasopressin type 2 receptor antagonist, which inhibits the binding of vasopressin and increases electrolyte-water clearance without activating the renin–angiotensin–aldosterone system or reducing the glomerular filtration rate [5, 6]. Therefore, TLV is an essential diuretic that is used as an add-on therapy to loop diuretics for managing patients with HF. The Efficacy of Vasopressin Antagonism in Heart Failure: Outcome Study with Tolvaptan (EVEREST) trial, a large-scale clinical study conducted in 2007, had reported results in two phases: short term and long term. Short-term improvements in HF signs and symptoms were observed. However, the long-term follow-up, which averaged 9.9 months, showed no improvements in all-cause mortality, cardiovascular mortality, and rehospitalization for HF [7, 8].

Approximately 10 years have passed, since tolvaptan became available for HF treatment in Japan, and the number of cases involving long-term administration of TLV to HF patients have increased. However, in the guidelines for HF by the Japanese Circulation Society, there are no clear rules for long-term administration of TLV and continuous administration of TLV to patients with class IIa HF and preserved ejection fraction (HFpEF). Few studies exist on the efficacy and safety of TLV, regardless of the ejection fraction (EF) of the patient it is administered to. In addition, few studies have compared its effects in patients with HFpEF and in patients with HF with reduced ejection fraction (HFrEF).

The number of HF patients administered TLV at our hospital has been increasing annually, and the number of patients who continue to receive TLV for more than 1 year has also been increasing. Therefore, we divided patients who had been using TLV for a long time at our hospital into two groups—HFpEF and HFrEF—with the goal of determining the patient group in whom long-term administration of TLV is beneficial for.

## Materials and methods

### Study design

This study was a retrospective single center trial. In total, 591 consecutive patients were admitted to our hospital and administered TLV for congestive HF between 2011 and 2018. We excluded patients who were administered TLV for < 1 year, lost to follow-up, had self-interrupted outpatient visits, or were prescribed medicine by other hospitals. Thus, 147 patients were ultimately enrolled in this study. HF was diagnosed on the basis of the criteria recommended in the Framingham Heart Study [9]. In this study, we defined HFpEF and HFrEF (including midrange EF) as left ventricular ejection fraction (LVEF) ≥ 50% and LVEF < 50%, respectively, determined by transthoracic echocardiography of patients with signs or symptoms of HF. The patients were classified into two groups—the HFpEF group (*n* = 77; 52.4%) and the HFrEF group (*n* = 70; 47.6%)—and followed up for a mean period of 2.7 years.

After discharge, the patients continued treatment with TLV. The dose was carefully increased or decreased in accordance with the patient’s condition. From January 2011 through December 2018, the patients were followed up at intervals of 1–2 months in the outpatient department of our hospital. The patients’ health status was checked during each follow-up and was recorded using electronic clinical records. Furthermore, all-cause death and hospitalization due to HF exacerbation were also verified.

This retrospective study was conducted using data from a large university hospital. The protocol used complied with the Declaration of Helsinki and was approved by our Institutional Ethics Committee, which waived the need for patient consent because of the retrospective nature of the study.

### Relevant factors

Hypertension was defined as a systolic blood pressure of ≥ 140 mmHg, a diastolic blood pressure of ≥ 90 mmHg, or the current use of antihypertensive agents. Atrial fibrillation (AF) was defined as paroxysmal AF or persistent AF, as documented by electrocardiography. Valvular heart disease was defined as moderate or severe aortic valve regurgitation and/or stenosis, moderate or severe mitral valve regurgitation and/or stenosis, and/or moderate or severe tricuspid valve regurgitation. During each echocardiographic study, the LVEF was calculated using the Teichholz method or the modified Simpson’s method. The severity of valvular heart disease was defined by quantitative measurements obtained by transthoracic echocardiography, such as flow velocity, pressure gradient, regurgitation volume, effective regurgitant orifice area, and regurgitation jet area. The doses of loop diuretics were converted to 20 mg furosemide, 30 mg azosemide, and 4 mg torasemide equivalents. Clinical data were obtained just before discharge once the hemodynamic conditions of the patients had stabilized. One year after initiating treatment, all parameters were remeasured and compared with the initial data.

### Endpoints

The primary endpoint was all-cause mortality. The secondary endpoint was cardiovascular mortality between the HFpEF group and the HFrEF group during the mean clinical follow-up period of 2.7 years. Furthermore, we conducted a stratified analysis (i.e., responder or nonresponder) on the basis of the response to TLV as reflected by urine osmolality. A responder had > 25% decrease in urine osmolality from a baseline > 350 mOsm/L for the first 4–6 h [10]. Urine osmolality was measured in 102 patients; of these, 40 were responders and 62 were nonresponders. We then subdivided each group into the HFpEF group and the HFrEF group and compared the patients’ characteristics, their all-cause mortality, and their cardiovascular mortality.

### Statistical analysis

SPSS, ver. 20.0 (SPSS, Inc., Chicago, IL, USA), was used for the statistical analyses. Continuous variables were expressed as the mean ± the standard deviation, and categorical variables were expressed as the number and percentage of patients. Survival and cardiac event-free curves were created using the Kaplan–Meier method. Differences in the survival and cardiac event-free rates between the groups were analyzed using the log-rank test. The relative risks in each group were calculated using Cox regression analyses. *P* values < 0.05 were statistically significant. Furthermore, we conducted a stratified analysis (i.e., responder or nonresponder), based on the response to TLV, as defined by urine osmolality.

## Results

**Fig. 1:**
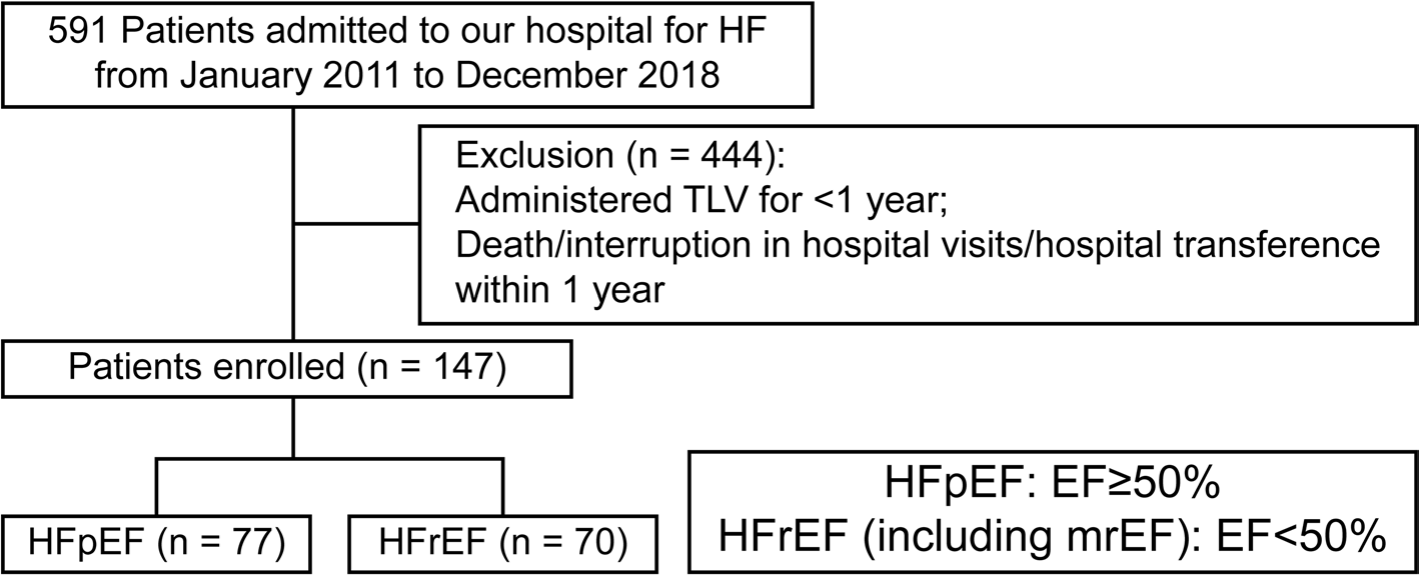
Patient selection process. This study was a retrospective, single center trial. In total, 591 consecutive patients were admitted to our hospital for CHF and were administered TLV between 2011 and 2018. After excluding 444 patients, 147 patients were ultimately enrolled in this study. We then divided the patients into HFpEF and HFrEF groups for further analysis. *EF* ejection fraction; *HF* heart failure; *HFpEF* heart failure with preserved ejection fraction; *HFrEF* heart failure with reduced ejection fraction; *mrEF* midrange EF; *TLV* tolvaptan

**Fig. 2:**
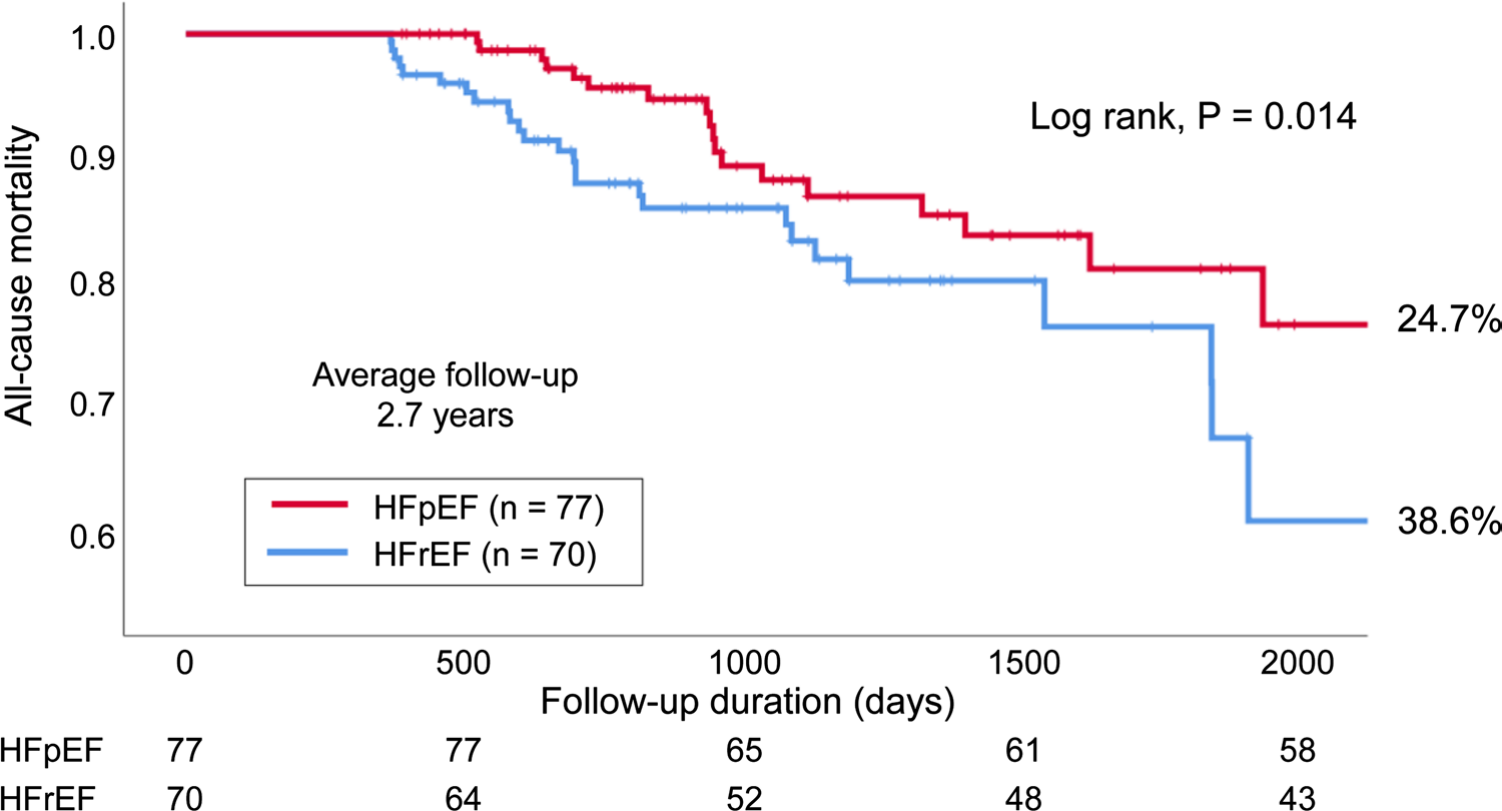
Kaplan–Meier curves for all-cause mortality in the HFpEF and HFrEF groups. During the mean follow-up period of 2.7 years, all-cause mortality occurred in 19 (24.7%) patients in the HFpEF group and 27 (38.6%) patients in the HFrEF group (log-rank, *P* = 0.014). HFpEF, heart failure with preserved ejection fraction; HFrEF, heart failure with reduced ejection fraction

**Fig. 3:**
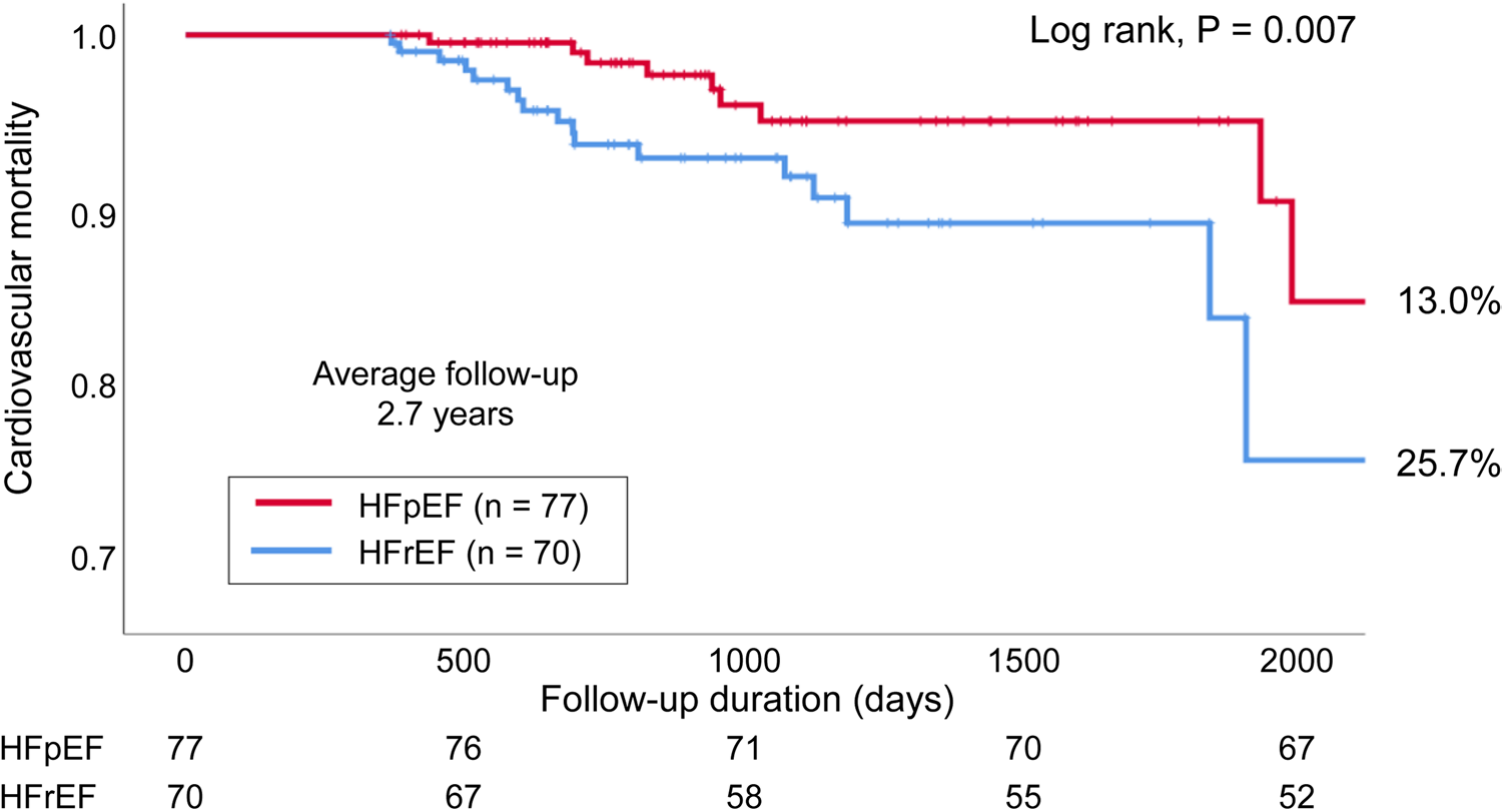
Kaplan–Meier curves for cardiovascular mortality in the HFpEF and HFrEF groups. Cardiovascular mortality occurred in 10 (13.0%) patients in the HFpEF group and 18 (25.7%) patients in the HFrEF group (log-rank, *P* = 0.007). *HFpEF* heart failure with preserved ejection fraction; *HFrEF* heart failure with reduced ejection fraction

Among the 147 patients, 77 (52.4%) patients had HFpEF and 70 (47.6%) patients had HFrEF (including HF with midrange EF) (Fig. 1). The baseline characteristics of the patients at the start of treatment with TLV are shown in Table 1. Compared with the HFrEF group, the HFpEF group included significantly older patients (71.3 ± 11.5 years vs. 77.7 ± 9.2 years, *P* < 0.01), more women (21.4% vs. 41.6%, *P* < 0.01), and more patients who had hypertensive heart disease, AF, and/or valvular heart disease as an underlying disease. However, the HFrEF group had more patients with dilated cardiomyopathy (DCM) and patients receiving β-blocker drugs or mineralocorticoid receptor antagonist (MRA) drugs. TLV and loop diuretic (furosemide-equivalent) doses at baseline and 1 year later did not differ between the two groups. The serum creatinine level was high but not significantly different between the HFpEF and HFrEF groups (1.55 ± 0.87 mg/dL vs. 1.78 ± 1.38 mg/dL; *P* = 0.225). According to the New York Heart Association (NYHA) classification, patients with NYHA II symptoms were significantly more common in the HFpEF group (85.7% vs. 58.6%, *P* < 0.01), and patients with NYHA III symptoms were significantly more common in the HFrEF group (13.0% vs. 38.6%, *P* < 0.01). Patients in the HFrEF group also commonly had a previous heart failure hospitalization history (39.0% vs. 58.6%, *P* = 0.017). Table 2 shows the patients’ backgrounds after 1 year of treatment with TLV. No significant differences were observed between the two groups in changes in *N*-terminal prohormone of brain natriuretic peptide (NT-proBNP) or in the concentrations of serum sodium, potassium, and creatinine over 1 year. At 1 year, compared with the HFpEF group, the HFrEF group showed a higher rate of initiation of β-blocker drugs and MRA, which are the standard treatments for HF. No significant difference in the use of loop diuretics or thiazide diuretics were observed between the two groups. The mean dose of TLV was 10 mg, which was the same dose in both groups. During the mean follow-up period of 2.7 years, all-cause mortality—the primary endpoint event—occurred in 19 (24.7%) patients in the HFpEF group and in 27 (38.6%) patients in the HFrEF group (log-rank, *P* = 0.014) (Fig. 2). Secondary endpoint events occurred in 10 (13.0%) patients in the HFpEF group and in 18 (25.7%) patients in the HFrEF group (log-rank, *P* = 0.007) (Fig. 3). However, there was no difference in the event-free survival rate between the two groups (57.1% vs. 70%, *P* = 0.108). Univariate Cox regression analysis of all-cause mortality, used to examine the patient population for which the long-term administration of TLV would be useful, suggested that HFpEF (hazard ratio [HR], 0.48; 95% confidence interval [CI], 0.26–0.87; *P* = 0.016), male sex (HR, 2.12; 95% CI, 1.02–4.40; *P* = 0.045), and an elevated serum creatinine level (HR, 1.50; 95% CI, 1.00–2.24; *P* = 0.049) were significant prognostic factors. Factors such as age, standard medication for HF, and valvular heart diseases were not found to be significant for the other baseline differences (Table 3). The multivariate Cox regression analysis, which adjusted for the significant factors in the univariate Cox regression analysis and age, suggested that HFpEF was the most significant prognostic factor (HR, 0.44; 95% CI, 0.23–0.86; *P* = 0.017).

**Table 1:** Patients’ characteristics: baseline

Variable	All (*n* = 147)	HFpEF (*n* = 77)	HFrEF (*n* = 70)	*P* value
Age (y)	74.6 ± 10.8	77.7 ± 9.2	71.3 ± 11.5	< 0.01
Male patient, *n* (%)	100 (68)	45 (58.4)	55 (78.6)	< 0.01
LVEF (%)	49.6 ± 19.5	65.8 ± 8.4	31.7 ± 10.5	< 0.01
NYHA II (%)	107 (72.8)	66 (85.7)	41 (58.6)	< 0.01
NYHA III (%)	37 (25.2)	10 (13.0)	27 (38.6)	< 0.01
NYHA IV (%)	3 (2.0)	1 (1.3)	2 (2.8)	0.515
Previous heart failure hospitalization (%)	71 (48.3)	30 (39.0)	41 (58.6)	0.017
Ischemic heart disease, *n* (%)	64 (43.5)	28 (36.4)	36 (51.4)	0.067
Hypertensive heart disease, *n* (%)	25 (17.0)	20 (26.0)	5 (7.1)	< 0.01
Dilated cardiomyopathy, *n* (%)	20 (13.6)	0 (0)	20 (28.6)	< 0.01
Hypertrophic cardiomyopathy, *n* (%)	7 (4.8)	6 (7.8)	1 (1.4)	0.071
Atrial fibrillation, *n* (%)	69 (46.9)	44 (57.1)	25 (35.7)	< 0.01
Valvular heart disease, *n* (%)	56 (38.1)	36 (46.8)	20 (28.6)	0.023
Hypertension, *n* (%)	80 (54.4)	50 (64.9)	30 (42.9)	< 0.01
Diabetes mellitus, *n* (%)	58 (39.5)	27 (35.1)	31 (44.3)	0.256
Dyslipidemia, *n* (%)	68 (46.2)	33 (42.9)	35 (50.0)	0.389
Hyperuricemia, *n* (%)	89 (60.5)	43 (55.8)	46 (65.7)	0.224
NT-proBNP (pg/mL)	7266	5451	7770	0.145
Na (mEq/L)	137.2 ± 6.1	137.3 ± 6.3	137.2 ± 5.9	0.889
K (mEq/L)	4.25 ± 0.61	4.20 ± 0.62	4.31 ± 0.60	0.274
Cre (mg/dL)	1.66 ± 1.14	1.55 ± 0.87	1.78 ± 1.38	0.225
β-blocker, *n* (%)	106 (72)	43 (55.8)	63 (90.0)	< 0.01
ACE-I or ARB, *n* (%)	104 (70.7)	52 (67.5)	52 (74.3)	0.372
MRA, *n* (%)	73 (49.7)	32 (41.6)	41 (58.6)	0.040
Furosemide, *n* (%)	64 (43.5)	38 (49.4)	26 (37.1)	0.096
Furosemide (mg)	33.6 ± 23.5	32.8 ± 18.0	34.8 ± 30.1	0.735
Azosemide, *n* (%)	54 (36.7)	22 (28.6)	32 (45.7)	0.031
Azosemide (mg)	48.1 ± 15.2	55.9 ± 10.5	42.7 ± 15.8	< 0.01
Torasemide, *n* (%)	29 (19.7)	17 (22.0)	12 (17.1)	0.456
Torasemide (mg)	5.31 ± 2.35	4.82 ± 2.13	6.0 ± 2.56	0.189
Trichlormethiazide, *n* (%)	17 (11.6)	10 (13.0)	7 (10.0)	0.575
Trichlormethiazide (mg)	1.32 ± 0.53	1.45 ± 0.60	1.14 ± 0.38	0.251
Loop diuretics, *n* (%)	127 (86.4)	68 (88.3)	59 (84.3)	0.800
Loop diuretics (furosemide-equivalent dose) (mg)	31.6 ± 25.4	32.1 ± 22.6	31.1 ± 28.4	0.480
TLV (initial dose) (mg)	4.97 ± 2.37	4.92 ± 2.04	5.04 ± 2.70	0.766
Urine osmolality (mOsm/kgH_2_O)	388.6 ± 115.1	383.1 ± 99.6	393.8 ± 128.5	0.625
Responder, *n* (%)	40/102 (39.2)	17/50 (34.0)	23/52 (44.2)	0.295

A responder is a patient who had > 25% decrease in urine osmolality from a baseline value of > 350 mOsm/L for the first 4–6 h. The values are presented as the mean ± the standard deviation, unless otherwise specified

*ACE-I* angiotensin-converting enzyme inhibitor; *ARB* angiotensin II receptor blocker; *Cre* creatinine; *HFpEF* heart failure with preserved ejection fraction; *HFrEF* heart failure with reduced ejection fraction; *K* potassium; *LVEF* left ventricular ejection fraction; *MRA* mineralocorticoid receptor antagonist; *Na* sodium; *NT-proBNP* N-terminal prohormone of brain natriuretic peptide; *NYHA* New York Heart Association; *TLV* tolvaptan; *y* years

**Table 2:** Patients’ characteristics: 1 year later

Variable	HFpEF (*n* = 77)		HFrEF (*n* = 70)		Intergroup *P* value
	Baseline	1 year later	Baseline	1 year later	
LVEF (%)	65.8 ± 8.4	65.0 ± 12.3	31.7 ± 10.5	32.8 ± 13.8	< 0.01
NT-proBNP (pg/mL)	5451	2759	7770	4217	0.224
Na (mEq)	137.3 ± 6.3	139.8 ± 3.89	137.2 ± 5.9	139.1 ± 4.89	0.331
K (mEq)	4.20 ± 0.62	4.47 ± 0.59	4.31 ± 0.60	4.59 ± 0.71	0.290
Cre (mg/dL)	1.55 ± 0.87	1.71 ± 0.89	1.78 ± 1.38	2.13 ± 1.81	0.072
ΔNT-proBNP (pg/mL)	–	−1519	–	−3481	0.559
ΔNa (mEq)	–	2.48 ± 7.06	–	1.91 ± 6.69	0.619
ΔK (mEq)	–	0.27 ± 0.76	–	0.27 ± 0.90	0.354
ΔCre (mg/dL)	–	0.16 ± 0.54	–	0.35 ± 1.11	0.184
β-blocker, *n* (%)	43 (55.8)	39 (50.6)	63 (90.0)	58 (82.9)	< 0.01
ACE-I or ARB, *n* (%)	52 (67.5)	54 (70.1)	52 (74.3)	53 (75.7)	0.451
MRA, *n* (%)	32 (41.6)	27 (35.1)	41 (58.6)	38 (54.3)	0.019
Furosemide, *n* (%)	38 (49.4)	39 (50.6)	26 (37.1)	28 (40.0)	0.198
Furosemide (mg)	32.8 ± 18.0	31.8 ± 23.2	34.8 ± 30.1	32.9 ± 29.8	0.870
Azosemide, *n* (%)	22 (28.6)	31 (40.3)	32 (45.7)	38 (54.3)	0.090
Azosemide (mg)	55.9 ± 10.5	48.9 ± 15.5	42.7 ± 15.8	44.2 ± 17.1	0.244
Torasemide, *n* (%)	17 (22)	15 (19.5)	12 (17.1)	11 (15.7)	0.553
Torasemide (mg)	4.82 ± 2.13	5.07 ± 2.25	6.0 ± 2.56	6.36 ± 2.16	0.153
Trichlormethiazide, *n* (%)	10 (13.0)	11 (14.3)	7 (10.0)	10 (14.3)	1.000
Trichlormethiazide (mg)	1.45 ± 0.60	1.14 ± 0.60	1.14 ± 0.38	1.75 ± 2.26	0.396
Loop diuretics, *n* (%)	68 (88.3)	72 (93.5)	59 (84.3)	66 (94.3)	0.845
Loop diuretics (furosemide-equivalent dose) (mg)	32.1 ± 22.6	34.2 ± 23.7	31.1 ± 28.4	34.1 ± 24.5	0.997
TLV (mg)	4.92 ± 2.04	10.1 ± 4.55	5.04 ± 2.70	10.3 ± 5.11	0.860

The delta (Δ) symbol indicates the change in the value 1 year later (i.e., the value at 1 year subtracted from the baseline value). The values are presented as the mean ± the standard deviation, unless otherwise specified

*ACE-I* angiotensin-converting enzyme inhibitor; *ARB* Angiotensin II receptor blocker; *Cre* creatinine; *HFpEF* heart failure with preserved ejection fraction; *HFrEF* heart failure with reduced ejection fraction; *K* potassium; *LVEF* left ventricular ejection fraction; *MRA* mineralocorticoid receptor antagonist; *Na* sodium; *NT-proBNP* N-terminal prohormone of brain natriuretic peptide; *TLV* tolvaptan

**Table 3:** Univariate and multivariate analysis for the predictors of all-cause mortality

	Univariate analysis		Multivariate analysis	
	HR (95% CI)	*P* value	HR (95% CI)	*P* value
Age	1.01 (0.98–1.04)	0.648	1.03 (0.99–1.07)	0.124
Male sex	2.12 (1.02–4.40)	0.045	1.76 (0.84–3.73)	0.137
HFpEF	0.48 (0.26–0.87)	0.016	0.44 (0.23–0.86)	0.017
NYHA II	0.64 (0.35–1.17)	0.146	–	–
NYHA III	1.47 (0.79–2.75)	0.222	–	–
Previous heart failure hospitalization	0.92 (0.51–1.66)	0.776	–	–
Atrial fibrillation	1.04 (0.58–1.88)	0.893	–	–
Hypertensive heart disease	0.36 (0.13–1.02)	0.053	–	–
Dilated cardiomyopathy	1.19 (0.47–3.03)	0.720	–	–
Valvular heart disease	1.23 (0.68–2.23)	0.487	–	–
Hypertension	0.57 (0.31–1.07)	0.082	–	–
Na (baseline)	1.02 (0.97–1.07)	0.444	–	–
Cr (baseline)	1.17 (0.89–1.53)	0.261	–	–
ΔNa	0.97 (0.93–1.02)	0.224	–	–
ΔCre	1.50 (1.00–2.24)	0.049	1.44 (0.99−2.09)	0.058
β blocker (baseline)	0.89 (0.47–1.68)	0.715	–	–
β blocker (1 year later)	0.64 (0.35–1.12)	0.141	–	–
MRA (baseline)	1.33 (0.73–2.42)	0.356	–	–
MRA (1 year later)	1.33 (0.74–2.40)	0.346	–	–
Furosemide dose (baseline)	0.98 (0.95–1.01)	0.174	–	–
Furosemide dose (1 year later)	1.01 (0.99–1.03)	0.158	–	–
Azosemide (1 year later)	1.07 (0.58–1.98)	0.836	–	–
Azosemide dose (1 year later)	0.97 (0.94–1.01)	0.114	–	–

The delta (Δ) symbol indicates the change in the value at 1 year later (i.e., the value at 1 year subtracted from the baseline value)

*CI* confidence interval; *Cre* creatinine; *HFpEF* heart failure with preserved ejection fraction; *HR* hazard ratio; *K* potassium; *MRA* mineralocorticoid receptor antagonist; *Na* sodium; *NT-proBNP* N-terminal prohormone of brain natriuretic peptide; *NYHA* New York Heart Association

A responder had more than 25% decrease in urine osmolality from the baseline > 350 mOsm/L for the first 4–6 h. We categorized the patients administered TLV into the responder group (*n* = 40; 39.2%) or the nonresponder group (*n* = 62; 60.8%). We further subdivided the responders and nonresponders into the HFpEF group or the HFrEF group (Online Resource 1). Among the responders, the HFrEF group included more patients with DCM, patients with a higher NT-proBNP, and patients with greater β blocker use than in the HFpEF group. Among the nonresponders, the HFrEF group included younger patients, more male patients, and more patients with ischemic heart disease and β blocker use than in the HFpEF group. The HFrEF group tended to have worse renal function and significantly higher potassium levels than did the HFpEF group. For both the responders and the nonresponders, the TLV dose after 1 year of treatment was not significantly different between the HFpEF and HFrEF groups (Online Resource 2). A comparison of all-cause mortality for the responders revealed no significant difference between the HFpEF group and the HFrEF group (23.5% vs. 21.7%, *P* = 0.897). However, for the nonresponders, all-cause mortality was significantly lower in the HFpEF group than in the HFrEF group (24.2% vs. 48.3%, *P* = 0.049). Cardiovascular mortality was not significantly different between the HFpEF group and the HFrEF group for both responders (5.9% vs. 17.4%, *P* = 0.288) and nonresponders (18.2% vs. 31.0%, *P* = 0.245) (Online Resource 3).

## Discussion

In the present study, we evaluated the long-term prognosis of patients administered TLV for > 1 year in the HFpEF and the HFrEF groups. During the follow-up (on average 2.7 years), the HFpEF group had significantly lower all-cause mortality and cardiovascular mortality than did the HFrEF group. Moreover, the male sex, HFpEF, and an elevated serum creatinine level were significant predictors of all-cause mortality in univariate Cox regression analysis. We also conducted a stratified analysis on the response to TLV. The findings revealed no differences in all-cause mortality between the HFpEF and HFrEF groups for responders, whereas all-cause mortality was significantly lower in the HFpEF group than in the HFrEF group for nonresponders.

In Japan, the clinical benefit of the short-term administration of TLV for HF in the acute phase has been reported in some studies [11, 12], and it has also been reported that TLV can be used relatively safely even in the elderly during the acute phase [13]. However, few reports exist regarding the long-term administration of TLV (especially for > 1 year). The EVEREST trial, which included short-term and long-term studies, revealed no significant differences between tolvaptan and a placebo in terms of all-cause mortality, cardiovascular mortality, or the time to readmission for HF during an average follow-up period of 9.9 months [7, 8]. The EVEREST study had not included patients with loop diuretic resistance. In addition, whether patients were or were not responders had not been reported. Therefore, some patients with HF may have rapidly improved without the administration of tolvaptan. The EVEREST trial also targeted patients with HFrEF. The current study enrolled only patients who had been clinically determined to need and receive the long-term administration of TLV. We then observed them longer than did previous studies of TLV, and we directly compared HFpEF and HFrEF [8, 14, 15].

Patients administered TLV often have chronic kidney disease (CKD). HF with CKD and worsening renal function (WRF) had been prevalent and associated with a strongly increased mortality risk in one study [16]. The efficacy of TLV administration is expected to improve the prognosis of HF patients with poor prognostic factors, such as CKD and WRF. Compared to furosemide, TLV administration in acute HF has been reported as advantageous with regard to renal function, hemodynamics, and neurohumoral factors [17, 18]. Uemura et al. [19, 20] reported that long-term administration of TLV to HF patients with CKD was relatively safe and effective when compared with treatment with conventional diuretic agents, whereas Nakao et al. [21] showed that long-term administration of TLV ameliorates the annual decline in estimated glomerular filtration rate in outpatients with HF. In another report [15], the long-term administration of TLV was reported to improve long-term prognosis in TLV responders, on the basis of increased urine volume, if the dose of loop diuretics were reduced with TLV therapy over 20 months. In this study, patients with CKD and loop diuretic resistance were included. No significant differences were observed in the increase of serum creatinine level in either group. The dose of loop diuretics remained largely unchanged after 1 year. In univariate analysis, an increased creatinine level was associated with worse all-cause mortality. This result is consistent with that in a previous report which had suggested that WRF leads to a worse prognosis [16].

Few reports exist regarding the long-term prognosis of TLV with a focus on HFpEF and HFrEF. Some studies [22, 23] have indicated that patients with HFpEF and HFrEF have a similar prognosis. Unlike HFrEF, no effective medication has been shown to improve the prognosis of HFpEF [24]. A previous report showed that the long-term administration of TLV did not reduce all-cause mortality but did reduce the readmission rate over 2 years [14]; the investigators had reported that long-term use of TLV tended to improve the 2-year all-cause mortality, irrespective of EF, among aquaporin-defined responders of TLV. In our study, multivariate analysis revealed that HFpEF was a significant predictor of all-cause mortality.

Several definitions exist for responders to TLV. We defined responders according to urine osmolality (i.e., > 25% decrease in urine osmolality from a baseline > 350 mOsm/L for the first 4–6 h) [10]. In this study, both groups contained approximately 40% responders. As a result of stratified analysis, on the basis of the response to TLV, there was a significant difference in all-cause mortality between the HFpEF and HFrEF groups among nonresponders. However, there was no significant difference in all-cause mortality between the two groups among responders; these findings are similar to those of a previous study by Imamura et al. [14]. On the basis of these results, the long-term use of TLV may be beneficial for patients with HFpEF, regardless of the responder status, whereas it may not be beneficial for nonresponders with HFrEF.

### Study limitations

This study had several limitations. First, this study had a relatively small number of enrolled patients and was of a retrospective nature carried out in a single center. Second, the discontinuation, increase, or decrease of TLV, the administration of other diuretics and the administration of other standard treatments for HF such as β-blockers was at the discretion of each doctor. Third, this study was restricted to patients who had been administered TLV for at least 1 year. The exclusion criteria may have led to the exclusion of many patients with a poor response to TLV and with more advanced heart failure. In general, patients with HFpEF are elderly, have many comorbidities, and are often admitted to other hospitals or institutions. Therefore, selection bias could have occurred and the prognosis of patients with HFpEF shown in this study may be limited. Fourth, this study included responders and nonresponders. The number of patients was small in the stratified analysis. Fifth, the mechanism supporting the results of this study was unclear. In other words, it was difficult to explain the mechanism underlying the difference in prognosis between the HFpEF and HFrEF groups of nonresponders. Future prospective studies should examine the benefits of TLV for HFpEF, which is a mixture of various pathological conditions.

In conclusion, the long-term administration of TLV may be more beneficial for patients with HFpEF than for patients with HFrEF. Moreover, among nonresponders to TLV, the long-term administration of TLV may not be beneficial for those with HFrEF. However, this conclusion should be confirmed through large-scale prospective randomized trials.

## Supplementary Information

Below is the link to the electronic supplementary material.

Supplementary file1 (TIF 450 KB)

Supplementary file2 (DOCX 34 KB)

## Data Availability

Because of the sensitive nature of the questions asked in this study, survey respondents were assured that their raw data would remain confidential and not be shared.
