# Supplementary figures and images for: Clinical features and long-term prognosis of patients with congestive heart failure taking tolvaptan: a comparison of patients with preserved and reduced left ventricular ejection fraction
Source: Heart Vessels. 2021 Oct 14;37(4):574–82. doi: 10.1007/s00380-021-01957-1 (PMC8917027; doi:10.1007/s00380-021-01957-1)

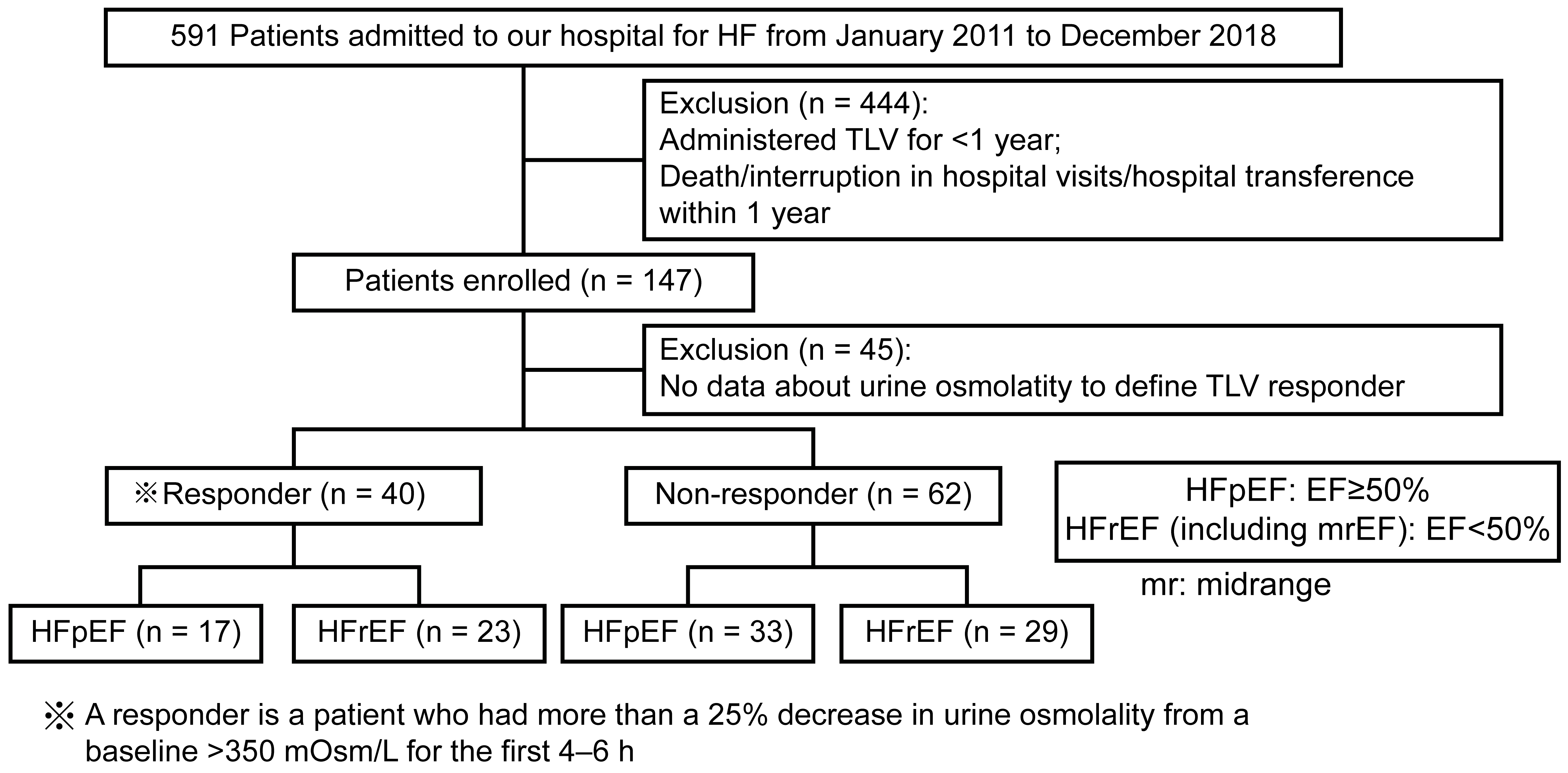

Supplement: Supplementary file 1 — Supplementary file1 (TIF 450 KB) [file 380_2021_1957_MOESM1_ESM.tif]
