# Supplementary material for: Clinical features and long-term prognosis of patients with congestive heart failure taking tolvaptan: a comparison of patients with preserved and reduced left ventricular ejection fraction
Source: Heart Vessels. 2021 Oct 14;37(4):574–82. doi: 10.1007/s00380-021-01957-1 (PMC8917027; doi:10.1007/s00380-021-01957-1)
Supplement: Supplementary file 2 — Supplementary file2 (DOCX 34 KB) [file 380_2021_1957_MOESM2_ESM.docx]

*Heart and Vessels*

**Clinical Features and Long-term Prognosis of Patients with Congestive Heart Failure Taking Tolvaptan: A Comparison Between Patients with Preserved and Reduced Left Ventricular Ejection Fraction**

Toshiki Seki, Yoshiaki Kubota, Junya Matsuda, Yukichi Tokita, Yu-ki Iwasaki, Wataru Shimizu

**Corresponding author:**

Yoshiaki Kubota, MD, PhD

ORCID: 0000-0001-7320-7641

Department of Cardiovascular Medicine, Nippon Medical School

1-1-5, Sendagi, Bunkyo-ku, Tokyo 113-0022, Japan

Phone No: +81-3-3822-2131; Fax No: +81-3-5685-0987; Email: [ykubota@nms.ac.jp](mailto:ykubota@nms.ac.jp)

**Online Resource 2.** Patients’ Characteristics: Responders and Nonresponders

| Variable | Responder (n=40) | | | P value | | Non-Responder (n=62) | | | | P value |
| --- | --- | --- | --- | --- | --- | --- | --- | --- | --- | --- |
|  | HFpEF (n=17) | HFrEF (n=23) | |  |  | HFpEF (n=33) | | HFrEF (n=29) | |  |
| Age (y) | 76.9 ± 6.1 | | 70.9 ± 12.8 | | 0.082 | | 79.2 ± 7.9 | | 72.7 ± 11.4 | 0.010 |
| Male, n (%) | 13 (76.5) | | 17 (73.9) | | 0.858 | | 16 (48.5) | | 22 (75.9) | 0.027 |
| LVEF (%) | 65.1 ± 8.0 | | 31.5 ± 10.9 | | <0.01 | | 66.1 ± 9.4 | | 33.2 ± 9.8 | <0.01 |
| Ischemic heart disease, n (%) | 6 (35.3) | | 10 (43.5) | | 0.612 | | 11 (33.3) | | 18 (62.1) | 0.024 |
| Hypertensive heart disease, n (%) | 5 (29.4) | | 1 (4.3) | | 0.028 | | 8 (24.2) | | 1 (3.4) | 0.02 |
| Dilated cardiomyopathy, n (%) | 0 (0) | | 9 (39.1) | | <0.01 | | 0 (0) | | 5 (17.2) | 0.012 |
| Hypertrophic cardiomyopathy, n (%) | 0 (0) | | 0 (0) | | - | | 5 (15.2) | | 1 (3.4) | 0.124 |
| Atrial fibrillation, n (%) | 11 (64.7) | | 8 (34.8) | | 0.063 | | 20 (60.6) | | 10 (41.7) | 0.135 |
| Valvular heart disease, n (%) | 4 (23.5) | | 4 (17.4) | | 0.642 | | 17 (51.5) | | 11 (37.9) | 0.291 |
| Hypertension, n (%) | 8 (47.1) | | 10 (43.5) | | 0.827 | | 23 (69.7) | | 13 (44.8) | 0.049 |
| Diabetes mellitus, n (%) | 6 (35.3) | | 11 (47.8) | | 0.441 | | 13 (39.4) | | 13 (44.8) | 0.671 |
| Dyslipidemia, n (%) | 7 (41.2) | | 12 (52.2) | | 0.504 | | 12 (36.4) | | 18 (62.1) | 0.044 |
| Hyperuricemia, n (%) | 8 (47.1) | | 13 (56.5) | | 0.565 | | 17 (51.5) | | 19 (65.5) | 0.272 |
| NT-proBNP (pg/mL) | 2564.2 | | 5397.7 | | 0.009 | | 7316.1 | | 11254.2 | 0.346 |
| Na (mEq/L) | 138.4 ± 7.2 | | 137.0 ± 7.7 | | 0.547 | | 136.3 ± 7.0 | | 137.3 ± 5.2 | 0.526 |
| K (mEq/L) | 4.25 ± 0.35 | | 4.17 ± 0.67 | | 0.648 | | 4.04 ± 0.69 | | 4.48 ± 0.46 | 0.005 |
| Cre (mg/dL) | 1.37 ± 0.51 | | 1.31 ± 0.37 | | 0.645 | | 1.49 ± 0.78 | | 2.15 ± 1.71 | 0.050 |
| β-blocker, n (%) | 8 (47.1) | | 19 (82.6) | | 0.006 | | 23 (69.7) | | 27 (93.1) | 0.020 |
| ACE-I or ARB, n (%) | 9 (52.9) | | 18 (78.3) | | 0.096 | | 23 (69.7) | | 24 (82.8) | 0.238 |
| MRA, n (%) | 10 (58.8) | | 12 (52.2) | | 0.685 | | 10 (30.3) | | 14 (48.3) | 0.152 |
| Furosemide, n (%) | 6 (35.3) | | 9 (39.1) | | 0.810 | | 16 (48.5) | | 10 (34.5) | 0.272 |
| Furosemide (mg) | 25.8 ± 16.9 | | 30.5 ± 16.7 | | 0.599 | | 38.8 ± 21.3 | | 40.0 ± 45.0 | 0.924 |
| Azosemide, n (%) | 5 (29.4) | | 11 (47.8) | | 0.251 | | 10 (30.3) | | 14 (48.3) | 0.152 |
| Azosemide (mg) | 54.0 ± 13.4 | | 39.6 ± 16.8 | | 0.114 | | 54.0 ± 12.6 | | 42.9 ± 15.4 | 0.074 |
| Torasemide, n (%) | 3 (17.6) | | 4 (17.4) | | 0.984 | | 8 (24.2) | | 6 (20.7) | 0.744 |
| Torasemide (mg) | 4.67 ± 3.06 | | 5.50 ± 1.92 | | 0.673 | | 4.50 ± 1.77 | | 6.33 ± 3.20 | 0.195 |
| Trichlormethiazide, n (%) | 3 (17.6) | | 2 (8.7) | | 0.410 | | 8 (24.2) | | 6 (20.7) | 0.646 |
| Trichlormethiazide (mg) | 0.83 ± 0.29 | | 1.00 ± 0 | | 0.495 | | 1.67 ± 0.52 | | 1.00 ± 0 | 0.035 |
| Tolvaptan (1 year later) (mg) | 9.04 ± 4.41 | | 9.09 ± 4.67 | | 0.974 | | 10.6 ± 4.61 | | 11.4 ± 5.41 | 0.522 |

A responder is a patient who had more than a 25% decrease in urine osmolality from a baseline value of >350 mOsm/L for the first 4–6 hours. The values are presented as the mean ± the standard deviation, unless otherwise specified.

ACE-I, angiotensin-converting enzyme inhibitor; ARB, angiotensin II receptor blocker; Cre, creatinine; HFpEF, Heart failure with preserved ejection fraction; HFrEF, heart failure with reduced ejection fraction; K, potassium; LVEF, left ventricular ejection fraction; MRA, mineralocorticoid receptor antagonist; Na, sodium; NT-proBNP, N-terminal prohormone of brain natriuretic peptide; y, years

**Online Resource 3.** All-cause Mortality and Cardiovascular Mortality in Responders and Nonresponders

| Variable | Responder (n=40) | | P value | Nonresponder (n=62) | | P value |
| --- | --- | --- | --- | --- | --- | --- |
|  | HFpEF (n=17) | HFrEF (n=23) |  | HFpEF (n=33) | HFrEF (n=29) |  |
| All-cause mortality, n (%) | 4 (23.5) | 5 (21.7) | 0.897 | 8 (24.2) | 14 (48.3) | 0.049 |
| Cardiovascular mortality, n (%) | 1 (5.9) | 4 (17.4) | 0.288 | 6 (18.2) | 9 (31.0) | 0.245 |

A responder is a patient who had more than a 25% decrease in urine osmolality from a baseline value of >350 mOsm/L for the first 4–6 hours.

HFpEF, heart failure with preserved ejection fraction; HFrEF, heart failure with reduced ejection fraction
